# Editorial: Novel Clinical Applications of Extracellular Vesicles

**DOI:** 10.3389/fimmu.2015.00381

**Published:** 2015-07-24

**Authors:** Matías Sáenz-Cuesta, María Mittelbrunn, David Otaegui

**Affiliations:** ^1^Multiple Sclerosis Unit, Biodonostia Health Research Institute, San Sebastián, Spain; ^2^Centro Nacional de Investigaciones Cardiovasculares (CNIC), Madrid, Spain

**Keywords:** extracellular vesicles, protocol standardization, clinical application, exosomes, biomarkers, immunotherapy, microvescicles, omics-technologies

Cells employ several modes of communication, from direct contact to paracrine signaling. Understanding what they are whispering one to another has long been a goal for immunologists. In this scenario, the potential of a newly discovered way of sending specific messages between cell types, extracellular vesicles (EVs), has started a revolution in the field.

During recent years, research on EVs has provided new insights into the pathophysiology of several diseases. EVs are membrane-bound particles secreted by almost all cell types. Depending on their biogenesis and size, they include exosomes, microparticles/microvesicles, and apoptotic bodies (1). Characteristically, EVs carry markers from the source cell membrane and contain genetic material, lipids, and proteins. They are known to play a role in cell-to-cell communication and to produce genotypic and phenotypic modifications in the target cell including antigen presentation, apoptosis induction, cellular activation, and differentiation (2). In particular, elevated concentrations of EVs have been found in many diseases including cancer (3), and autoimmune (4) and cardiovascular diseases (5). Most research on EVs is focused on their characterization, identifying mechanisms of action, and their potential use as biomarkers, with some studies investigating therapeutic applications. In relation to the promising results obtained in the EV field; however, there are some technical issues concerning standardization to be resolved and these are being addressed by researchers. Further, even though EVs have a growing future as biomarkers, drug delivery systems, or therapeutic targets, there remain milestones to be achieved on the path to their clinical application. This research topic aims to provide a forum for the discussion of current and novel clinical applications of EVs, opening new avenues in this field.

We focus the topic on two closely related fields where EVs have produced the most impact, namely, cancer and immunology. First, de Toro et al. have summarized the current roles of EVs in physiological and pathological and physiological states, including neurodegenerative, cardiovascular, and immune diseases (6). They conclude with an interesting section on the potential applications of EVs in diagnosis and therapy.

In relation to cancer, Benito-Martín et al. have reviewed the functions of innate immune-derived EVs in relation to modification of microenvironment and the control of tumor progression (7). The authors also provide a detailed description of the role of EVs derived from innate immune cells in specific cancers (colorectal cancer, osteosarcoma, neuroblastoma, and neurofibromatosis-1-related tumors). They point to the lack of information on these EVs under physiological conditions.

The proposal suggested by Carvalho and Oliveira is the use of EVs as a “liquid biopsy” overcoming the limited information provided by a single fragment of a tumor (8). They also assert that EVs may be circulating biomarkers with a potential role in the detection of the early stages of cancer. However, they note that the identification of EVs and characterization of their cargo should be carefully analyzed depending on whether they have been isolated in a human cancer sample or in a cancer cell-line culture. Finally, they propose a longitudinal approach involving the sampling of EVs over the course of a disease.

Moving specifically to the biomarker area, Gámez-Valero et al. have conducted a comprehensive review of urine-derived EV candidates for monitoring kidney diseases (9). These authors affirm that urinary EVs reflect the state of urinary system; however, depending on the isolation protocol used, published data are not always comparable. In relation to this, they detail current and some novel methods for isolating EVs from urine and provide a clear review of urinary EV biomarkers by type of kidney disease. They also stress the urgent need for a consensus on methods and for these to then be applied to larger cohorts.

In an attempt to respond to the aforementioned suggestion, Sáenz-Cuesta et al. have compared five different EV isolation protocols (including the standard ultracentrifugation approach), starting with blood and urine samples as these are the most readily available, and several types of analysis (flow cytometry, nanoparticle tracking analysis, and electronic microscopy, among others) (10). The authors propose an interesting workflow for the study of EVs in a hospital setting, taking into account the facilities of a non-specialized core laboratory and based on an easy and quick medium-speed centrifugation protocol.

The application of the study of EVs in a daily clinical setting requires suitable technologies and quality controls that could be managed by the hospital itself or delegated to specific facilities. Regarding the facilities provided by culture platforms, Aiastui posit that they should offer a basic level of quality control for the production of large quantities of EVs in good manufacturing practice conditions (11). For that, close collaboration between clinicians from hospitals, the biotech industry, and basic researchers is necessary to turn what is currently an idea into a product. As an example, Aiastui suggest that according to current requirements, culture platforms should become EV sample quality control services before EV products are applied as a therapy.

Research into EV cargo is continuously growing thanks to the spread of these new technologies. One of the most recently applied omic approaches, metabolomics, is focused on metabolites, cytosolic small molecules up to 1 kDa. Palomo et al. analyze the benefits of adding this to the spectrum of omic approaches applied to EVs, discussing which platform is the most suitable and also warning about the importance of additional background controls (e.g., exosome-depleted media analysis) (12).

Finally, Romagnoli et al. hypothesize about the possibility of using dendritic cell (DC)-derived EVs as cancer immunotherapy enhancing tumor antigenicity (13). They demonstrate the boost effect of DC-derived EVs in primed T-cell activation against an adenocarcinoma cell line and conclude that both DCs and tumor cells became more immunogenic after the incorporation of specific EVs.

It is the desire of the editors that this topic should help to connect all interested authors and readers, and join our efforts to understand what and how the cells are whispering.

## Conflict of Interest Statement

The authors declare that the research was conducted in the absence of any commercial or financial relationships that could be construed as a potential conflict of interest.
